# Multi-Scale 3D Cephalometric Landmark Detection Based on Direct Regression with 3D CNN Architectures

**DOI:** 10.3390/diagnostics14222605

**Published:** 2024-11-20

**Authors:** Chanho Song, Yoosoo Jeong, Hyungkyu Huh, Jee-Woong Park, Jun-Young Paeng, Jaemyung Ahn, Jaebum Son, Euisung Jung

**Affiliations:** 1 Medical Device Development Center, Daegu-Gyeongbuk Medical Innovation Foundation (K-MEDI Hub), Daegu 41061, Republic of Korea; chsong@kmedihub.re.kr (C.S.); hkhuh@kmedihub.re.kr (H.H.); jp@kmedihub.re.kr (J.-W.P.); 2 Daegu-Gyeongbuk Research Division, Electronics and Telecommunications Research Institute (ETRI), Daegu 42994, Republic of Korea; yoosoojeong@etri.re.kr; 3 Department of Oral and Maxillofacial Surgery, Samsung Medical Center, Sungkyunkwan University School of Medicine, Seoul 06351, Republic of Korea; jypaeng@gmail.com (J.-Y.P.); jaemyung.ahn@samsung.com (J.A.)

**Keywords:** cephalometric landmark detection, 3D convolutional neural network (CNN), cephalometric analysis

## Abstract

Background: Cephalometric analysis is important in diagnosing and planning treatments for patients, traditionally relying on 2D cephalometric radiographs. With advancements in 3D imaging, automated landmark detection using deep learning has gained prominence. However, 3D imaging introduces challenges due to increased network complexity and computational demands. This study proposes a multi-scale 3D CNN-based approach utilizing direct regression to improve the accuracy of maxillofacial landmark detection. Methods: The method employs a coarse-to-fine framework, first identifying landmarks in a global context and then refining their positions using localized 3D patches. A clinical dataset of 150 CT scans from maxillofacial surgery patients, annotated with 30 anatomical landmarks, was used for training and evaluation. Results: The proposed method achieved an average RMSE of 2.238 mm, outperforming conventional 3D CNN architectures. The approach demonstrated consistent detection without failure cases. Conclusions: Our multi-scale-based 3D CNN framework provides a reliable method for automated landmark detection in maxillofacial CT images, showing potential for other clinical applications.

## 1. Introduction

Cephalometric analysis involves the quantitative measurements of anatomical landmarks in the craniofacial region and plays an important role in patient diagnosis and surgical planning. Traditionally, cephalometric analysis has been conducted with 2D cephalometric radiographs using lateral view analysis. However, with advancements in computer vision technology, 3D imaging techniques such as computed tomography (CT) and cone beam computed tomography (CBCT) are being applied, leading to research on 3D cephalometric analysis.

Cephalometric analysis is conducted by detecting craniofacial anatomical landmarks and performing linear and angular measurements between the landmarks. In the process of landmark analysis, the accurate detection of anatomical landmarks is an essential step for accurate analysis. However, conventional landmark detection involves time-consuming manual measurement by medical professionals, which are prone to measurement variability [1,2]. To address these issues, several studies reported an automated detection of anatomical landmarks using deep learning technologies, which improved the accuracy of cephalometric landmark detection.

Automatic 2D cephalometric measurement focuses on landmark detection in lateral radiographs, with various methods developed to enhance accuracy. Yao et al. applied self-supervised learning with a multi-task U-Net, leveraging cascade cosine similarity maps and pseudo-labels [1]. Chen et al. developed a semi-supervised approach using shape-regulated self-training with U-Net and the spatial configuration network (SCN) [2]. Zhu et al. proposed a universal anatomical landmark detection model consisting of a local network and a global network [3]. Yao et al. proposed a cephalometric landmark detection network comprising a global module and a local module based on a U-Net architecture [4].

While 2D cephalograms remain commonly used in medical practice, which contains clear limitations, such as the superimposition of anatomical structures, 3D cephalometric imaging (e.g., CT, CBCT) provides detailed access to complex craniofacial and maxillomandibular structures, enabling precise analysis. However, the increased dimensionality of 3D imaging introduces more trainable parameters and network complexity in deep learning-based approaches. Consequently, training with 3D images presents challenges, and various approaches have been developed to address these difficulties in 3D cephalometric landmark detection.

A method for automatic 3D landmark detection is the 2.5-dimensional approach, which involves learning slices of 3D images. Ma et al. proposed a patch-based deep learning model that uses 2D convolution on extracted patches to achieve 3D landmark extraction [5]. Dot et al. developed a model incorporating a coarse-to-fine structure into the SCN [6]. This approach detects landmark regions of interest (ROIs) through coarse detection, and then performs fine detection using heatmaps within each ROI to extract 3D landmarks.

A widely used approach for 3D landmark detection is to localize landmark coordinates using regression methods with the 3D convolutional filter [7]. Zheng et al. proposed a method combining 3D CNNs with a bootstrapped detector, leveraging Haar wavelet features for landmark detection [8]. Yun et al. proposed a coarse-to-fine structure integrated with a variational auto-encoder (VAE) to detect 93 3D landmarks [9]. Liu et al. reported a multi-stage CNN model that employs a 3D U-Net for cephalometric landmark detection and bone segmentation, treating these tasks as voxel classification [10]. Nishimoto et al. proposed a multi-phased deep regression model that uses progressively smaller cropped voxels to achieve refined localization [11].

Recent studies have introduced 3D detection methods to simultaneously address 3D localization and classification problems. These studies commonly employ two-stage networks, which include a region proposal network (RPN) and Bounding Box Recognition, to identify regions containing landmarks and estimate the coordinates. Lang et al. reported a method for automatic landmark detection applying a 3D R-CNN [12]. The approach applied a 3D Mask R-CNN with a coarse-to-fine structure considering three stages of low- and high-resolution images. A root/leaf structured loss function was introduced to group anatomical landmarks by dividing them into nine geometric regions, and the method was validated using CBCT images from 49 patients. Chen et al. proposed a landmark detection method using 3D Faster R-CNN and 3D U-Net [13]. By applying a coarse-to-fine structure, the method first identifies approximate locations within CBCT images and then uses a multi-scale U-Net to detect landmark coordinates. Heatmap regression was used to train the U-Net, and the proposed method was validated using 80 sets of clinical CBCT data. Sahlsten et al. implemented a deep learning method using a stacked hourglass network to automate the identification of 3D cephalometric landmarks [14]. This single-stage model, built on a stacked hourglass network architecture, incorporated 3D convolutional layers optimized for computational efficiency. Model performance was evaluated based on the mean Euclidean distance and success detection rate across 46 cephalometric landmarks, using a dataset of 309 CBCT scans.

Previous studies have proposed methods for automatically detecting anatomical landmarks in 3D medical images. These methods are based on training 3D convolutional filters in deep learning and aim to improve accuracy through the application of auto-encoders, detection, segmentation, or stacked hourglass network architectures. Studies applying RCNN-type networks have shown relatively high accuracy by incorporating region proposal networks or heatmap regression. This study employed a stacked hourglass network architecture that achieved an accuracy of less than 2 mm. However, these approaches can still result in failure cases when detecting cephalometric anatomical landmarks, which may limit their applicability in clinical settings. In contrast, direct regression methods can produce trained landmarks without failure cases, but the accuracy required for clinical application has not yet been reported.

In this paper, a method for 3D cephalometric landmark detection by applying a coarse-to-fine structure into direct regression is presented. The proposed method employs multi-scale direct regression with a 3D CNN to extract 3D local patches for each landmark from the global maxillofacial region, followed by refining the landmark coordinates within local patches. The optimal base network was identified through performance comparisons of previous 3D CNN architectures for direct regression, and the proposed method was validated using 150 clinical datasets. The contributions of this study are summarized as follows:

(1) Performance comparison of 3D CNN architectures for landmark direct regression;
(2) Development of an automatic cephalometric landmark detection network using multi-scale direct regression;
(3) Validation using 150 sets of maxillofacial clinical data (CT).

## 2. Methods

### 2.1. Method Overview

**Figure 1:**
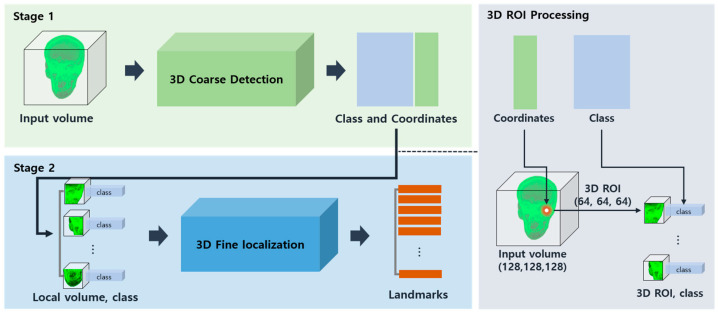
Diagram of multi-scale cephalometric landmark detection architecture. Stage 1 involves coarse detection to identify coordinates and classes from the entire input volume, followed by generating local volumes through 3D region of interest (ROI) processing. Stage 2 focuses on fine localization using these local volumes and classes.

A multi-scale cephalometric landmark detection method based on direct regression with 3D CNN aims to enhance detection accuracy through a hierarchical learning strategy on multi-scale volumes. This method includes coarse detection to extract the class and coordinates of all landmarks from the global input volume, 3D region of interest (ROI) processing to transform these results into local volumes, and fine localization to precisely determine the landmark coordinate within a local volume, as illustrated in Figure 1.

Stage 1 involves coarse detection to determine the coordinates and classes of landmarks across the entire input volume, resulting in the identification of classes and coordinates for 30 anatomical landmarks. Subsequently, 3D ROI processing is performed to generate local volumes corresponding to each landmark. Stage 2 focuses on fine localization, using the local volumes and classes obtained from Stage 1. Sub-sampled local volumes at a scale of 64 × 64 × 64 with the corresponding class are used to precisely localize landmark coordinates at the local scale. Further details on the networks employed in each stage are discussed in Section 2.5.

### 2.2. Data Description

CT or CBCT imaging is a widely accepted modality for modern maxillofacial diagnosis and treatment planning. Clinical maxillofacial CT images from 150 patients between 1 January 2010 and 31 July 2022 were collected retrospectively at Samsung Medical Center, with approval from the Institutional Review Board of the Department of Oral and Maxillofacial Surgery, Samsung Medical Center, Seoul, Korea (SMC 2022-08-091-001). Data from patients with severe diseases, those still in growth, and those with craniofacial anomalies were excluded to ensure the dataset was suitable for normal oral and maxillofacial diagnosis and treatment planning.

Collected data were anonymized and processed to create the medical imaging dataset. The dataset comprised only the 3D reconstructed volume data of the skull, excluding facial soft tissues. Volumes were generated by segmenting the maxillofacial skull regions, and all patient metadata were removed for anonymization. For supervised learning, 3D anatomical landmark data were established as ground truths. The ground truth consisted of 30 anatomical landmarks: (11apex, 13, 16, 21apex, 23, 26, A_point, ANS, B, Ba, CoL, CoR, Gn, GoL, GoR, L1, Lt_FZS, Menton, Na, Nasaltip, OrL, OrR, PNS, PoL, PoR, Pog, Rt_FZS, S, Sp, U1). Clinical experts at Samsung Medical Center manually identified 30 anatomical landmarks used in maxillofacial surgical planning, and 3D coordinates for these landmarks were annotated on each anonymized maxillofacial volume.

### 2.3. Data Preprocessing

**Figure 2:**
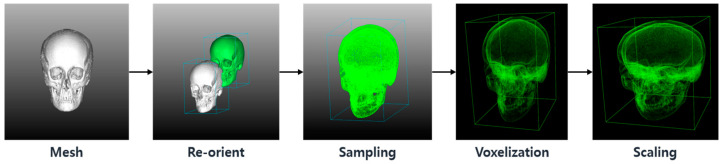
Steps for data preprocessing to convert volumetric CT data into normalized voxel data.

Data preprocessing is required to convert maxillofacial volumetric data (or segmented mesh data) into a normalized format suitable for 3D CNN application. In prior studies [7,8,9,10,11,12,13], voxel data have been used as the input format for the training of inference of 3D CNNs. In this study, maxillofacial data were provided in mesh format (e.g., STL) due to the internal policy for personal medical information protection. Consequently, a preprocessing algorithm was developed and applied to prepare the maxillofacial volume data for CNN training, as shown in Figure 2.

The preprocessing algorithm was developed using Open3D [15] and the Visualization Toolkit [16] open-source libraries. To standardize the coordinate system for the maxillofacial volumetric data, the origin was set at the front bottom left point of a bounding cube encompassing the 3D data. To expedite the voxelization process, uniform sampling was applied to select 50,000 points. The sampled volume was voxelized using the Open3D library, and the data were normalized to the scale of 128 × 128 × 128 voxels.

### 2.4. Three-Dimensional CNN Architectures

Convolutional Neural Networks (CNNs) are deep learning architectures optimized for feature extraction and regression in spatially structured data, such as images. Consisting of multiple layers (e.g., convolutional, pooling, Fully Connected Layers), CNNs automatically extract and learn features from low to high levels, making them effective for object recognition and classification.

Three-dimensional CNNs extend conventional CNNs to handle three-dimensional data. By using 3D Convolution and 3D pooling, these networks can learn from additional spatial or temporal dimensions and are applied in areas like robotics, autonomous driving, and medical imaging (e.g., CT, MRI). Three-dimensional CNNs have also been employed in diagnostics, particularly for predicting protein–ligand interactions [17,18,19] and assessing the binding affinity between RNA and small molecules [20]. Notably, 3D CNNs have shown promising results in medical imaging tasks such as tumor detection, classification, and tissue segmentation using 3D images such as CT, MRI, and PET [21,22,23,24].

**Figure 3:**
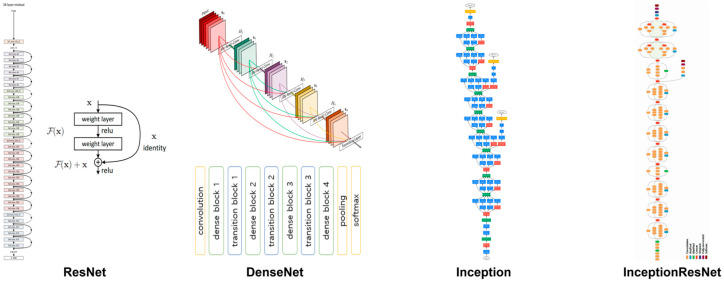
Convolutional Neural Network (CNN) architectures including ResNet, DenseNet, Inception, and InceptionResNet.

In this study, 3D CNNs were applied to solve detection tasks in 3D maxillofacial images via voxel-wise regression. Three-dimensional CNN architectures, including ResNet, DenseNet, Inception, and InceptionResNet, as shown in Figure 3, were trained on 3D maxillofacial images to assess landmark detection accuracy. We trained and evaluated each network using the maxillofacial clinical dataset and compared the landmark detection accuracy to select the optimal network for 3D maxillofacial landmark detection.

#### 2.4.1. ResNet Architecture

ResNet is a model that introduced residual learning to overcome the degradation of learning performance associated with increased network depth [25]. Typically, deepening a neural network to solve complex problems leads to a significant increase in trainable parameters, causing the gradient vanishing problem. Residual learning addresses this by having each layer learn a residual function *F*(*x*) = *H*(*x*) − *x* instead of the direct mapping *H*(*x*). By approximating the residuals to zero, optimization becomes easier, allowing identity mapping without adding parameters or computational complexity. This approach mitigates the gradient vanishing issue, enabling the training of deeper networks. ResNet is available in configurations with various depths, such as 50, 101, or 152 layers, and its effectiveness has been validated using datasets like ImageNet and CIFAR-10.

#### 2.4.2. DenseNet Architecture

DenseNet (Densely Connected Convolutional Networks) is a CNN architecture designed to maximize feature map reuse by connecting all layers in a feed-forward fashion [26]. In DenseNet, each layer receives input from all preceding layers and passes its output to all subsequent layers, creating a dense connectivity pattern within dense blocks. This approach enhances feature integration and reuse, differing from ResNet by connecting features rather than summing them. A network with *L* layers will have *L* (*L* + 1)/2 direct connections. To handle the increasing number of feature maps, the growth rate *k* is set as a hyper-parameter, typically *k* = 12, allowing for narrower layers and fewer parameters. Dense blocks reduce network complexity and alleviate gradient vanishing through feature reuse. By stacking dense blocks, structural expansion is possible, leading to improved performance as network depth increases. The effectiveness of the architecture has been validated using object recognition benchmarks such as CIFAR-10, CIFAR-100, SVHN, and ImageNet.

#### 2.4.3. Inception Architecture

Inception networks aim for high performance and computational efficiency by incorporating dimensional reduction and parallel structures [27]. The key element of the Inception architecture is the Inception module, which simultaneously utilizes multiple filter sizes, such as 1 × 1, 3 × 3, and 5 × 5 convolution layers, combined with pooling layers. This design allows for balanced expansion in both depth and width, facilitating the extraction and reuse of diverse features through parallel convolutional filters. The use of 1 × 1 convolutions within these parallel structures also aids in dimensional reduction, enhancing computational efficiency. Inception-v3 introduced additional optimization techniques based on the Inception module. By stacking 3 × 3 convolution layers, as in VGGNet [28], an effective 7 × 7 convolution layer was created, further improving computational efficiency. Label smoothing was also employed to reduce overfitting and enhance generalization performance. Inception networks have shown high performance on large-scale image classification benchmarks like ImageNet, demonstrating their versatility across various computer vision tasks.

#### 2.4.4. InceptionResNet Architecture

InceptionResNet is a network model that combines the Inception module, which uses multiple filter sizes simultaneously, with identity mapping via shortcut connections [29]. The main feature of the InceptionResNet architecture is the addition of residual connections following the Inception module, which uses parallel combinations of filters. This setup allows the network to perform residual learning on the Inception module’s parallel structures. By adjusting the activation scaling value of the residual connections, the model’s training stability is improved, alleviating gradient vanishing and increasing the learning speed. InceptionResNet has demonstrated high performance on the ImageNet dataset and has shown that applying residual connections can significantly enhance training speed.

### 2.5. Proposed Architecture

In order to achieve multi-scale 3D cephalometric landmark detection based on direct regression with the 3D CNN, we sought to experimentally identify the suitable base architecture for landmark detection from existing 3D CNN structures. A comparative experiment was conducted to identify the optimal CNN architecture for cephalometric landmark detection, with detailed results provided in Section 3. Among the existing CNN architectures, the 169-layer DenseNet demonstrated the lowest error in landmark detection experiments using the maxillofacial clinical dataset. Consequently, DenseNet169 was selected as the base architecture for developing the multi-scale CNN structure in this study.

**Figure 4:**
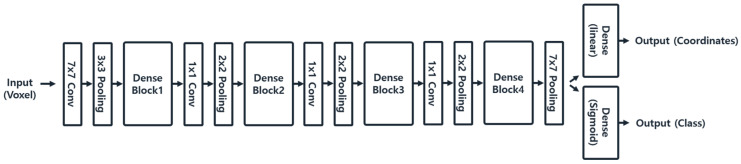
DenseNet169-based multi-output learning model for coarse detection.

**Figure 5:**
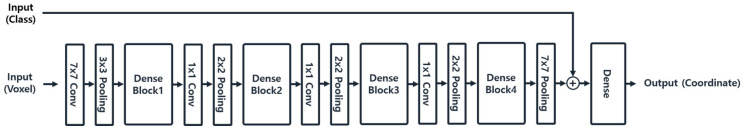
DenseNet169-based multi-input learning model for fine localization.

For the implementation of multi-scale direct regression, a coarse detection architecture was designed to detect the coordinates and classes of all landmarks across the entire volume, while a fine localization architecture was developed to precisely localize individual landmark coordinates within local regions, as depicted in Figure 4 and Figure 5. This study used 30 landmarks for training and inference. In the coarse detection, all landmarks were regressed simultaneously to generate 30 local volumes and classes. The fine localization then used these local volumes and classes to localize landmark coordinates at a local scale.

#### 2.5.1. Coarse Detection

Coarse detection utilizes a multi-output learning model to extract the coordinates and classes of all 30 landmarks from a scale of 128 × 128 × 128 input volume. The model is designed to learn the coordinates and classes of the 30 landmarks simultaneously. To address the regression problem of landmark coordinates, mean absolute error (MAE) loss and mean squared error (MSE) metrics are employed. For classifying landmark classes, binary cross-entropy loss and binary accuracy metrics are used. The loss weights for the multi-output model are set to 0.9 for regression and 0.1 for classification to ensure balanced learning for both outputs. With this network configuration, the coarse detection structure processes a scale of 128 × 128 × 128 input volume and outputs the 30 × 3 coordinates and 30 × 30 classes for the 30 landmarks.

#### 2.5.2. Three-Dimensional Region of Interest (ROI) Processing

Coarse detection provides the coordinates of 30 anatomical landmarks and their corresponding classes from the maxillofacial image. In this study, local patches were generated for multi-scale learning based on the coarse detection results by creating 3D ROIs using the inferred landmark coordinates. To create these 3D ROIs, each of the 30 landmarks identified in the 128 × 128 × 128 input volume was used as a central coordinate. A local volume with dimensions of 64 along the x, y, and z axes was then defined around each landmark. As a result, 30 sets of 64 × 64 × 64 local volumes were generated from an input volume, each centered on a landmark, to support local learning.

#### 2.5.3. Fine Localization

The objective of fine localization is to precisely localize the coordinates of landmarks within a local volume that includes the landmark, focusing on the local-scale features. To accomplish this, a multi-input learning model was employed, using the local volume and corresponding class from the coarse detection results to infer the final landmark coordinates. The fine localization network uses two inputs—a 64 × 64 × 64 local volume and the corresponding class—and it outputs 3 × 1 fine coordinates. The local volume is processed through dense blocks and then concatenated with the class input before the final dense layer for learning. Since the task is to predict landmark coordinates, a regression approach was applied using mean absolute error (MAE) as the loss function and mean squared error (MSE) as the metric. With this network configuration, the fine localization network utilizes the multi-inputs of local volume and class to localize coordinates within the local region. The resulting coordinates are initially specific to the local region but are converted to the global scale to serve as the final landmark coordinates.

## 3. Experimental Results

This section presents a comparative experiment with existing 3D CNN architectures and the performance evaluation of the proposed method. We assessed 3D CNN models, including ResNet [25], DenseNet [26], Inceptionv3 [27], and Inception-ResNetV2 [29], for their performance in 3D cephalometric landmark detection. Additionally, we tested the landmark detection capabilities of the proposed method.

The 150 sets of maxillofacial clinical CT images from Samsung Medical Center, mentioned in Section 2.2, were normalized and used for network training and evaluation. Of the 150 datasets, 120 were used for training and 30 were used for validation and testing.

Each CNN model was trained with the following hyper-parameters: Adam optimizer, MSE loss function, batch size of 41,000 epochs, and a learning rate of 0.001. The proposed method involved two training phases: coarse (using Adam optimizer, MAE-BinaryCrossEntropy loss function, batch size of 41,000 epochs, learning rate of 0.001) and fine (using Adam optimizer, MAE loss function, batch size of 15,1000 epochs, learning rate of 0.001).

Experiments were conducted on a Precision 7920 workstation (Dell, Round Rock, TX, USA) with Microsoft Windows 10 Pro for Workstations, an Intel Xeon Gold 5218R CPU, and an NVIDIA RTX A6000 GPU, using Python 3.9.16, Keras 2.10.0, Open3D 0.17.0 [15], and Visualization Toolkit 9.2.6 [16].

Table 1 compares the network parameters, accuracy, and inference time for maxillofacial landmark detection. Accuracy is measured as the mean and variance of the root-mean-square error (RMSE) between network predictions and the ground truth. Parameters indicate the number of trainable parameters, and times represent the average inference time per prediction. Regarding the accuracy of existing CNN architectures, Inception-ResNetV2 had the highest error at 2.978 ± 0.493 mm, while DenseNet169 had the lowest error at 2.464 ± 0.382 mm. The proposed method achieved an error of 2.238 ± 0.364 mm, reducing the error by approximately 0.74 mm compared to Inception-ResNetV2 and by about 0.226 mm compared to DenseNet169. Table 2 shows the RMSE for each of the 30 maxillofacial landmarks across different networks. The proposed method achieved the lowest error of 1.457 ± 0.674 mm for landmark GN and the highest error of 3.038 ± 1.862 mm for landmark GoL.

## 4. Discussion

In this study, a multi-scale 3D CNN architecture for automatic cephalometric landmark detection is proposed. This approach employs a coarse-to-fine strategy, performing global-scale detection followed by local-scale localization to automatically extract landmarks. The coarse-to-fine architecture enables the use of both global-scale and local-scale features, allowing for localization refinement by subdividing the volume scale. In the proposed method, multi-output learning and multi-input learning structures were applied. This approach enabled feature learning across the entire maxillofacial volume to regress landmark coordinates and classes, while also allowing for local refinement within a subdivided volume scale in the local region. Unlike previous landmark detection studies [5,6,7,8,9,10,11,12,13], to the best of the authors’ knowledge, this is the first research integrates both a coarse-to-fine structure and direct regression.

To validate the proposed method, 150 sets of clinical CT data and 30 anatomical landmarks identified by clinical experts were used. The network’s ability to detect cephalometric landmarks was evaluated by comparing the RMSE between the network-predicted coordinates and those determined by experts. Comparative experiments with existing CNN architectures revealed that the proposed method achieved 2.238 mm of the lowest average accuracy and 0.364 mm of the lowest standard deviation. In comparison to conventional CNN models, our results show an accuracy improvement of approximately 25% over Inception-ResNetV2, 23% over InceptionV3, 20% over ResNet152, and 9% over DenseNet169. In the proposed method, DenseNet169 was applied as the backbone network for feature reuse, suggesting that the application of a multi-stage structure contributed to a 9% improvement in accuracy. An individual error comparison for the 30 cephalometric anatomical landmarks showed that the proposed method achieved the lowest error for 24 landmarks, followed by DenseNet201, which recorded the lowest error for two landmarks. DenseNet169, ResNet152, ResNet101, and ResNet50 each recorded the lowest error for one landmark. Among the 3D CNN structures, deep learning architectures designed for feature reuse (e.g., DenseNet) were found to potentially lower the accuracy of landmark detection.

Objective accuracy comparisons across 3D cephalometric landmark detection studies are challenging due to variations in clinical data (e.g., the number of images and landmarks). Some state-of-the-art studies [12,13] reported ~1 mm accuracy with 80 CBCT scans and 18 landmarks, and ~2 mm accuracy with 49 CBCT scans and 105 landmarks. These studies have the advantage of generating ROIs through the network, enabling precise localization refinement. However, they use region-proposal-based approaches, resulting in failure cases, with reported accuracies excluding these failure cases. In contrast, the proposed method, utilizing multi-scale direct regression for landmark inference, achieved a ~2.24 mm error without failure cases.

In the design of the multi-scale detection architecture, the trainable parameter was achieved at 39,056,605, with an average inference time of 3.835 s. In the process of designing and training deep learning networks, trainable parameters are directly related to computational cost, training time, and network complexity. From this perspective, the DenseNet169 backbone network was chosen in the proposed method for its efficient feature reuse capability, which minimizes the number of trainable parameters and enables deeper learning without adding a significant computational burden. The two-stage learning strategy increases network complexity; although the trainable parameters have increased compared to the base architecture (DenseNet169), it remains lower than that of the shallower ResNet50 architecture. The inference time, representing the time taken for a single prediction, was higher compared to existing 3D CNN architectures. Since the goal of this study is to apply the method in pre-operative procedures such as patient diagnosis or surgical planning, prioritizing higher accuracy over reduced inference time will improve its clinical applicability.

For clinical applicability, cephalometric analysis generally requires an accuracy below 2 mm [1,2,3], indicating the need for further accuracy enhancements in future research. Specifically, achieving a detection accuracy below 2 mm is important for clinical acceptance, but the absence of detection failures is also crucial to maintain clinical viability. In this study, we aimed to achieve landmark detection accuracy without any failure cases to ensure this viability. Furthermore, the capability to detect 30 landmarks without failure cases suggests potential clinical utility, especially if post-processing steps are introduced to allow clinicians to refine landmarks.

In deep learning, model generalization is important for applicability across diverse clinical environments, making the construction of high-quality, massive datasets an important task. Building those clinical datasets for research presents practical challenges, such as the volume of data accumulated at hospitals and patient protection policies. Particularly in medical fields with relatively low demand, building extensive datasets can remain a challenging issue. In this research, due to these limitations, 150 CT datasets were used to implement the proposed method. However, in future studies, methods such as federated learning, which enables data collaboration across multiple hospitals, may provide a viable solution for these limitations.

Some limitations remain. First, although the multi-scale design enhances accuracy, it also increases inference time due to the multi-stage detection process. For applications in broader fields, especially during surgical procedures, further optimization may be needed to reduce computation time. Secondly, this study focused only on the skull region of the maxillofacial volume, excluding surrounding soft tissues to ensure patient privacy. This approach required additional preprocessing steps for anonymization and limited the ability to learn features of soft tissues.

Considering recent advancements in deep learning model structures, there remains potential to further enhance accuracy in future research. In this study, a 3D CNN architecture and a coarse-to-fine strategy were applied to improve landmark detection accuracy. The coarse-to-fine structure has been reported to enhance localization accuracy [12,13], a result also demonstrated in this study for cephalometric landmark detection. However, studies have been published that advance conventional CNNs, and future research could consider applying Vision Transformer (ViT) [30] or stacked hourglass architecture [14] to further improve accuracy.

This study focused on using only the skull from maxillofacial images, requiring segmentation to isolate the skull for deep learning network learning. To ensure patient privacy, soft tissues surrounding the skull were removed during the anonymization process and were not used in this study. Future research aims to develop deep learning networks that can directly utilize clinical data by implementing training servers within healthcare institutions and exporting only the trained models (e.g., deep network weights).

## 5. Conclusions

In this paper, a multi-scale image-based CNN architecture was proposed for automatic 3D cephalometric landmark detection. This method applies direct regression in multi-scale learning, enabling landmark detection without failure cases. Experimental results with clinical data indicate that the proposed approach enhances cephalometric landmark detection performance compared to existing CNN methods. Although the proposed method requires prior segmentation of the skull volume, future research aims to develop algorithms that can be directly used in clinical settings by operating training servers within healthcare institutions. We expect that the concept of the proposed method has the potential for application in surgical planning and medical analysis involving maxillofacial imaging.

## Figures and Tables

**Table 1:** Comparison of trainable parameters, accuracy, and inference time for landmark detection of the proposed method and existing CNN architectures.

	Parameters	Accuracy (mm)	Time (s)
Inception-ResNetV2	67,647,802	2.978 ± 0.493	0.246
Inceptionv3	34,299,770	2.917 ± 0.501	0.225
ResNet50	46,377,501	2.787 ± 0.545	0.118
ResNet101	85,475,869	2.754 ± 0.473	0.142
ResNet152	117,680,669	2.783 ± 0.490	0.302
DenseNet201	25,732,762	2.505 ± 0.391	0.172
DenseNet121	11,418,522	2.467 ± 0.435	0.232
DenseNet169	18,850,970	2.464 ± 0.382	0.334
DenseNet169 + Coarse-to-fine (**Proposed**)	39,056,605	2.238 ± 0.364	3.835

**Table 2:** The accuracy for each of the 30 cephalometric landmark coordinates across the proposed method and existing CNN architectures.

	Inception ResNetV2	Inceptionv3	ResNet50	ResNet101	ResNet152	DenseNet201	DenseNet121	DenseNet169	Proposed
11apex	2.497 ± 0.922	2.620 ± 1.177	2.302 ± 1.136	2.087 ± 0.956	2.379 ± 1.081	1.959 ± 0.889	2.068 ± 0.873	1.925 ± 0.876	1.815 ± 0.945
13	2.412 ± 1.090	2.473 ± 1.085	2.268 ± 0.936	2.330 ± 0.889	2.450 ± 1.106	2.145 ± 1.098	2.197 ± 0.944	2.153 ± 0.940	2.028 ± 0.913
16	3.221 ± 1.180	3.343 ± 1.270	2.865 ± 1.146	2.823 ± 1.388	3.201 ± 1.463	2.882 ± 1.226	2.956 ± 1.121	2.973 ± 1.247	2.875 ± 1.208
21apex	2.477 ± 0.865	2.506 ± 1.255	2.221 ± 0.931	2.130 ± 0.976	2.475 ± 0.900	1.947 ± 0.776	1.951 ± 0.819	1.838 ± 0.785	1.953 ± 0.731
23	2.748 ± 1.226	3.008 ± 1.524	2.574 ± 1.065	2.572 ± 1.159	2.803 ± 1.265	2.278 ± 1.166	2.345 ± 1.228	2.286 ± 1.129	2.053 ± 1.074
26	3.097 ± 1.147	3.395 ± 1.452	2.996 ± 1.300	2.969 ± 1.271	3.243 ± 1.652	2.886 ± 1.288	2.986 ± 1.192	2.978 ± 1.357	2.900 ± 1.244
A-point	2.484 ± 1.001	2.595 ± 1.062	2.265 ± 0.953	2.306 ± 0.995	2.319 ± 0.843	2.083 ± 0.860	2.133 ± 0.913	2.179 ± 0.954	2.106 ± 0.985
ANS	2.778 ± 1.356	3.003 ± 1.367	2.611 ± 1.205	2.869 ± 1.422	2.394 ± 1.188	2.416 ± 1.325	2.414 ± 1.379	2.495 ± 1.322	2.656 ± 1.676
B	2.933 ± 1.408	2.824 ± 1.255	2.537 ± 1.407	2.746 ± 1.328	2.550 ± 1.198	2.821 ± 1.148	2.354 ± 1.255	2.515 ± 1.207	2.041 ± 1.118
Ba	3.271 ± 1.822	2.427 ± 1.132	3.102 ± 1.661	3.124 ± 1.631	3.051 ± 1.742	2.680 ± 1.504	2.418 ± 1.226	2.683 ± 1.386	2.131 ± 1.135
CoL	3.444 ± 1.073	3.383 ± 1.522	3.661 ± 1.861	3.821 ± 1.767	3.847 ± 1.285	3.274 ± 1.445	3.319 ± 1.300	2.907 ± 1.373	2.832 ± 1.393
CoR	3.618 ± 1.532	3.351 ± 1.486	3.549 ± 1.435	3.543 ± 1.236	3.326 ± 1.459	3.060 ± 1.306	2.946 ± 1.117	2.862 ± 1.035	2.860 ± 1.126
Gn	3.189 ± 1.438	2.650 ± 1.440	2.367 ± 1.219	2.373 ± 0.961	2.319 ± 0.800	2.196 ± 1.171	1.884 ± 1.075	1.938 ± 0.927	1.457 ± 0.674
GoL	4.448 ± 2.079	4.608 ± 1.949	4.104 ± 2.034	4.184 ± 2.197	4.104 ± 2.099	3.567 ± 2.101	3.660 ± 1.779	3.666 ± 1.866	3.038 ± 1.862
GoR	4.213 ± 1.623	3.740 ± 1.476	3.875 ± 1.459	3.741 ± 1.731	3.417 ± 1.574	3.473 ± 1.579	3.386 ± 1.654	3.469 ± 1.745	2.664 ± 1.444
L1	2.645 ± 1.051	2.803 ± 1.029	2.932 ± 0.981	2.900 ± 0.979	2.924 ± 0.963	2.730 ± 0.877	2.502 ± 0.937	2.734 ± 0.942	2.291 ± 0.934
Lt. FZS	2.281 ± 1.198	2.654 ± 1.282	2.592 ± 1.180	2.419 ± 1.020	2.367 ± 1.254	2.053 ± 0.843	2.040 ± 0.853	2.120 ± 0.845	1.860 ± 0.891
Menton	3.048 ± 1.743	2.687 ± 1.249	2.358 ± 1.294	2.292 ± 0.884	2.253 ± 0.951	2.091 ± 1.107	2.042 ± 1.079	1.935 ± 0.981	1.533 ± 0.746
Na	2.297 ± 1.193	2.394 ± 1.102	2.228 ± 1.073	2.197 ± 0.912	2.218 ± 0.991	1.732 ± 0.930	1.708 ± 0.918	1.767 ± 0.992	1.699 ± 0.805
Nasal tip	3.001 ± 1.598	3.009 ± 1.184	2.574 ± 1.273	2.657 ± 1.163	2.756 ± 1.211	2.662 ± 1.125	2.795 ± 1.407	2.710 ± 1.400	2.689 ± 1.535
OrL	2.967 ± 1.505	3.093 ± 1.484	2.864 ± 1.418	2.559 ± 1.264	2.863 ± 1.424	2.644 ± 1.090	2.639 ± 1.028	2.633 ± 1.129	2.318 ± 1.211
OrR	3.183 ± 1.184	3.092 ± 1.210	2.601 ± 0.841	2.876 ± 1.166	2.785 ± 1.325	2.572 ± 0.996	2.624 ± 1.062	2.664 ± 1.003	2.312 ± 0.980
PNS	2.889 ± 1.574	2.698 ± 1.851	2.826 ± 1.787	2.828 ± 1.740	2.855 ± 1.741	2.687 ± 1.656	2.604 ± 1.636	2.804 ± 1.438	2.597 ± 1.451
PoL	3.570 ± 1.553	3.031 ± 1.229	3.583 ± 1.591	3.295 ± 1.649	3.321 ± 1.554	3.041 ± 1.300	2.830 ± 1.413	2.819 ± 1.216	2.774 ± 1.251
PoR	3.310 ± 1.419	2.704 ± 1.114	3.402 ± 1.627	2.865 ± 1.199	2.886 ± 1.338	2.459 ± 1.169	2.525 ± 1.024	2.658 ± 0.973	2.253 ± 0.978
Pog	3.045 ± 1.369	2.681 ± 1.247	2.467 ± 1.169	2.508 ± 0.966	2.603 ± 0.721	2.324 ± 0.937	1.968 ± 0.984	2.018 ± 0.847	1.687 ± 0.813
Rt. FZS	2.838 ± 1.253	2.594 ± 1.061	2.140 ± 1.025	2.371 ± 1.516	2.307 ± 1.172	2.140 ± 1.173	2.102 ± 1.085	1.991 ± 1.091	1.747 ± 1.102
S	2.232 ± 1.374	2.606 ± 1.300	2.544 ± 1.213	2.319 ± 1.311	2.381 ± 1.305	1.971 ± 1.119	2.039 ± 1.046	1.872 ± 1.109	1.845 ± 0.940
Sp	2.780 ± 1.808	2.879 ± 1.241	2.662 ± 1.551	2.537 ± 1.426	2.699 ± 1.589	2.149 ± 1.288	2.297 ± 1.370	2.122 ± 1.308	2.033 ± 1.374
U1	2.430 ± 1.016	2.659 ± 1.062	2.532 ± 0.970	2.375 ± 1.053	2.382 ± 1.047	2.224 ± 1.062	2.272 ± 0.969	2.201 ± 0.897	2.091 ± 0.872

## Data Availability

The original contributions presented in the study are included in this article, and further inquiries can be directed to the corresponding authors.

## References

[1] Yao Q., Quan Q., Xiao L., Kevin Zhou S. (2021). One-shot medical landmark detection. Medical Image Computing and Computer Assisted Intervention–MICCAI 2021, Proceedings of the 24th International Conference, Strasbourg, France, 27 September–1 October 2021.

[2] Chen R., Ma Y., Liu L., Chen N., Cui Z., Wei G., Wang W. (2022). Semi-supervised anatomical landmark detection via shape-regulated self-training. Neurocomputing.

[3] Zhu H., Yao Q., Xiao L., Zhou S.K. (2021). You only learn once: Universal anatomical landmark detection. Medical Image Computing and Computer Assisted Intervention–MICCAI 2021, Proceedings of the 24th International Conference, Strasbourg, France, 27 September–1 October 2021.

[4] Yao J., Zeng W., He T., Zhou S., Zhang Y., Guo J., Tang W. (2022). Automatic localization of cephalometric landmarks based on convolutional neural network. Am. J. Orthod. Dentofac. Orthop..

[5] Ma Q., Kobayashi E., Fan B., Nakagawa K., Sakuma I., Masamune K., Suenaga H. (2020). Automatic 3D landmarking model using patch-based deep neural networks for CT image of oral and maxillofacial surgery. Int. J. Med. Robot. Comput. Assist. Surg..

[6] Dot G., Schouman T., Chang S., Rafflenbeul F., Kerbrat A., Rouch P., Gajny L. (2022). Automatic three-dimensional cephalometric landmarking via deep learning. J. Dent. Res..

[7] Kang S.H., Jeon K., Kim H.-J., Seo J.K., Lee S.-H. (2020). Automatic three-dimensional cephalometric annotation system using three-dimensional convolutional neural networks: A developmental trial. Comput. Methods Biomech. Biomed. Eng. Imaging Vis..

[8] Zheng Y., Liu D., Georgescu B., Nguyen H., Comaniciu D. (2015). 3D deep learning for efficient and robust landmark detection in volumetric data. Medical Image Computing and Computer-Assisted Intervention—MICCAI 2015, Proceedings of the 18th International Conference, Munich, Germany, 5–9 October 2015.

[9] Yun H.S., Jang T.J., Lee S.M., Lee S.-H., Seo J.K. (2020). Learning-based local-to-global landmark annotation for automatic 3D cephalometry. Phys. Med. Biol..

[10] Liu Q., Deng H., Lian C., Chen X., Xiao D., Ma L., Chen X., Kuang T., Gateno J., Yap P.-T. (2021). SkullEngine: A multi-stage CNN framework for collaborative CBCT image segmentation and landmark detection. Machine Learning in Medical Imaging, Proceedings of the 12th International Workshop, MLMI 2021, Held in Conjunction with MICCAI 2021, Strasbourg, France, 27 September 2021.

[11] Nishimoto S., Saito T., Ishise H., Fujiwara T., Kawai K., Kakibuchi M. (2023). Three-Dimensional Craniofacial Landmark Detection in Series of CT Slices Using Multi-Phased Regression Networks. Diagnostics.

[12] Lang Y., Wang L., Yap P.-T., Lian C., Deng H., Thung K.-H., Xiao D., Yuan P., Shen S.G., Gateno J. (2019). Automatic detection of craniomaxillofacial anatomical landmarks on CBCT images using 3D mask R-CNN. Graph Learning in Medical Imaging, Proceedings of the First International Workshop, GLMI 2019, Held in Conjunction with MICCAI 2019, Shenzhen, China, 17 October 2019.

[13] Chen X., Lian C., Deng H.H., Kuang T., Lin H.-Y., Xiao D., Gateno J., Shen D., Xia J.J., Yap P.-T. (2021). Fast and accurate craniomaxillofacial landmark detection via 3D faster R-CNN. IEEE Trans. Med. Imaging.

[14] Sahlsten J., Järnstedt J., Jaskari J., Naukkarinen H., Mahasantipiya P., Charuakkra A., Vasankari K., Hietanen A., Sundqvist O., Lehtinen A. (2024). Deep learning for 3D cephalometric landmarking with heterogeneous multi-center CBCT dataset. PLoS ONE.

[15] Zhou Q.-Y., Park J., Koltun V. (2018). Open3D: A modern library for 3D data processing. arXiv.

[16] Schroeder W., Martin K., Lorensen B. (2006). The Visualization Toolkit.

[17] Jiménez J., Skalic M., Martinez-Rosell G., De Fabritiis G. (2018). K deep: Protein–ligand absolute binding affinity prediction via 3d-convolutional neural networks. J. Chem. Inf. Model..

[18] Li Y., Rezaei M.A., Li C., Li X. (2019). DeepAtom: A framework for protein-ligand binding affinity prediction. Proceedings of the 2019 IEEE International Conference on Bioinformatics and Biomedicine (BIBM).

[19] Wang Y., Qiu Z., Jiao Q., Chen C., Meng Z., Cui X. (2021). Structure-based protein-drug affinity prediction with spatial attention mechanisms. Proceedings of the 2021 IEEE International Conference on Bioinformatics and Biomedicine (BIBM).

[20] Sun S., Gao L. (2024). Contrastive pre-training and 3D convolution neural network for RNA and small molecule binding affinity prediction. Bioinformatics.

[21] Chen H., Dou Q., Yu L., Qin J., Heng P.-A. (2018). VoxResNet: Deep voxelwise residual networks for brain segmentation from 3D MR images. NeuroImage.

[22] Milletari F., Navab N., Ahmadi S.-A. (2016). V-net: Fully convolutional neural networks for volumetric medical image segmentation. Proceedings of the 2016 Fourth International Conference on 3D Vision (3DV).

[23] Kompanek M., Tamajka M., Benesova W. (2019). Volumetric data augmentation as an effective tool in mri classification using 3d convolutional neural network. Proceedings of the 2019 International Conference on Systems, Signals and Image Processing (IWSSIP).

[24] Alakwaa W., Nassef M., Badr A. (2017). Lung cancer detection and classification with 3D convolutional neural network (3D-CNN). Int. J. Adv. Comput. Sci. Appl..

[25] He K., Zhang X., Ren S., Sun J. Deep residual learning for image recognition. Proceedings of the IEEE Conference on Computer Vision and Pattern Recognition.

[26] Huang G., Liu Z., Van Der Maaten L., Weinberger K.Q. Densely connected convolutional networks. Proceedings of the IEEE Conference on Computer Vision and Pattern Recognition.

[27] Szegedy C., Vanhoucke V., Ioffe S., Shlens J., Wojna Z. Rethinking the inception architecture for computer vision. Proceedings of the IEEE Conference on Computer Vision and Pattern Recognition.

[28] Simonyan K., Zisserman A. (2014). Very deep convolutional networks for large-scale image recognition. arXiv.

[29] Szegedy C., Ioffe S., Vanhoucke V., Alemi A. Inception-v4, inception-resnet and the impact of residual connections on learning. Proceedings of the AAAI Conference on Artificial Intelligence.

[30] Tamhane A., Mida T.E., Posner E., Bouhnik M. (2022). Colonoscopy landmark detection using vision transformers. MICCAI Workshop on Imaging Systems for GI Endoscopy.

